# Therapeutic Drug Monitoring in Special Circumstances in Inflammatory Bowel Disease

**DOI:** 10.3390/jcm14227956

**Published:** 2025-11-10

**Authors:** Sebastian Povlsen, Kamal Patel, Xavier Roblin, Konstantinos Papamichael, Sailish Honap

**Affiliations:** 1 Department of Gastroenterology, St George’s University Hospital NHS Foundation Trust, London SW17 0QT, UK; 2 Department of Gastroenterology, Saint-Etienne University Hospital, 42055 Saint-Etienne, France; 3 Department of Gastroenterology, Center for Inflammatory Bowel Diseases, Beth-Israel Deaconess Medical Center, Harvard Medical School, Boston, MA 02215, USA; 4 School of Immunology and Microbial Sciences, King’s College London, London SE1 9RT, UK

**Keywords:** therapeutic drug monitoring, inflammatory bowel disease, ulcerative colitis, Crohn’s disease

## Abstract

Inflammatory bowel disease, encompassing ulcerative colitis and Crohn’s disease, is characterised by chronic immune-mediated inflammation and variable treatment response. Loss of drug efficacy due to underexposure, pharmacokinetic variability, and immunogenicity remains a key challenge. Therapeutic drug monitoring, using drug levels and anti-drug antibody measurements, is an important strategy for optimising the treatment of inflammatory bowel disease. It helps ensure adequate dosing and can distinguish between pharmacokinetic and mechanistic drug failure. Most evidence pertains to infliximab and adalimumab. Multiple factors influence drug pharmacokinetics, affecting both target drug levels and the doses required to achieve them. These include inflammatory burden, bodyweight, age, disease phenotype, and route of administration, all of which are important considerations for individualising treatment in inflammatory bowel disease. This narrative review explores how special clinical situations—acute severe ulcerative colitis, perianal fistulising Crohn’s disease, hypoalbuminaemia, extremes of body composition, pregnancy, paediatrics, and advanced age—alter drug pharmacokinetics and influence the utility and interpretation of therapeutic drug monitoring in inflammatory bowel disease.

## 1. Introduction

Inflammatory bowel disease (IBD) is a collective term for a group of lifelong, immune-mediated inflammatory diseases, broadly comprising ulcerative colitis (UC) and Crohn’s disease (CD) [1,2]. Treatment for IBD is often hampered by poor drug persistence, with approximately one third of patients needing to change their advanced therapy after a year [3]. Suboptimal persistence reflects a mix of underexposure, pharmacokinetic (PK) variability, and immunogenicity, which collectively drive loss of response and treatment failure. Therapeutic drug monitoring (TDM), including drug levels and anti-drug antibody (ADA) assessment, is established for infliximab (IFX), adalimumab (ADM), and thiopurines to extend treatment durability and prevent avoidable relapse from underdosing or ADA formation to anti-tumour necrosis factor alpha (TNFα) drugs [4,5]. TDM helps distinguish PK failure (amenable to dose optimisation or immunomodulator co-therapy) from mechanistic failure (prompting a switch out of class), enabling more rational, cost-based decisions and reducing unnecessary drug cycling.

In clinical practice, TDM can be applied reactively, when primary non-response or secondary loss of response is suspected, or proactively, with scheduled measurement of trough concentrations to maintain targets associated with remission. Some evidence supports a proactive versus reactive approach to TDM [6,7], although several studies were not designed to directly answer this question and methodological limitations temper certainty [8,9]. Beyond thiopurines and anti-TNFα agents, the evidence base for TDM remains contentious and not part of routine practice at present [10]. The PANTS study showed that low drug concentrations at week 14 were the only independent risk factor for primary non-response to IFX and ADM in CD, with remission associated with drug levels of 7 mg/L and 12 mg/L, respectively [11]. Major societies endorse TDM for IFX, ADM, and thiopurines; uniquely, ESPGHAN currently recommends early proactive TDM in paediatric CD, while others deem evidence insufficient to prefer it over reactive use [12,13,14,15,16]. Regardless of strategy, practical barriers still impede TDM implementation [17].

Multiple patient variables influence drug levels and targets and include disease phenotype, disease severity, body composition, pregnancy, age, and administration route [10,18]. Evidence around these factors continues to evolve, reshaping how TDM is applied and how targets are set in specific clinical contexts. This narrative review synthesises current evidence across these scenarios and highlights evidence gaps and research priorities to advance routine, patient-centred TDM.

## 2. Methods

In August 2025, MEDLINE (via PubMed) was searched using the following terms: “therapeutic drug monitoring” AND (“inflammatory bowel disease” OR “ulcerative colitis” OR “Crohn’s disease”). Additional searches included: “acute severe ulcerative colitis,” “albumin,” “fistula,” “perianal,” “body composition,” “weight,” “obesity,” “adipose,” “pregnancy,” “paediatric,” “age,” “elderly,” and “subcutaneous.” Reference lists of included studies were hand-searched for further articles. We included randomised controlled trials (RCTs), post hoc analyses of RCTs, meta-analyses, retrospective and prospective cohort studies, cross-sectional studies, and population pharmacokinetic studies. Narrative and systematic review articles were also accepted and their reference lists searched for further studies.

## 3. Disease Severity and Phenotype

### 3.1. Acute Severe Ulcerative Colitis

Acute severe ulcerative colitis (ASUC) is a life-threatening manifestation of UC, classically defined by Truelove and Witts criteria (≥6 bloody stools/day plus systemic toxicity), requiring urgent inpatient management. The biologic era ushered in by IFX, one of only two therapies repeatedly showing efficacy in ASUC (the other is ciclosporin), has markedly reduced colectomy rates, particularly in the short term [19,20]. Despite this, up to 20% of patients require colectomy during their first admission, which increases on subsequent admissions [20,21]. Consequently, optimisation of inpatient medical rescue therapy is still needed. To date, optimal dosing regimens and the role of TDM in this situation have not clearly been demonstrated. In patients with moderate to severe UC, post hoc analysis of the landmark ACT1 and ACT2 trials showed that patients in the lowest quartile for serum IFX concentration were less likely to achieve clinical response, remission, and mucosal healing, irrespective of the IFX dose given [22]. Increased IFX clearance also predicts colectomy in ASUC [23]. This supports a target-concentration approach to overcome increased drug clearance and improve outcomes.

**Figure 1:**
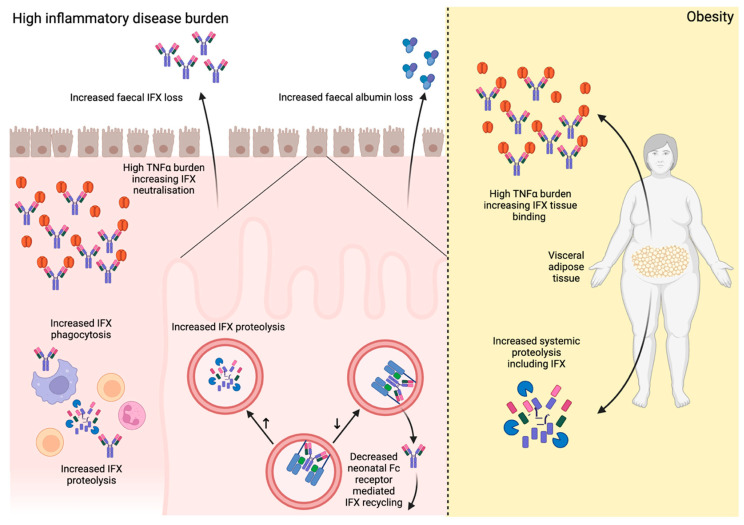
Mechanisms of infliximab clearance. In disease states with a high inflammatory burden, there is increased damage of the intestinal epithelium, leading to increased faecal loss of infliximab (IFX) and albumin, which is associated with low IFX levels. There is also increased tumour necrosis factor alpha (TNFα) in the tissue, which then binds and neutralises a greater proportion of IFX, acting as a ‘TNFα sink’. Through increased intestinal epithelial cell damage, there is also reduced neonatal Fc receptor availability, thus reducing the recycling of IFX to circumvent lysosomal proteolysis, resulting in increased degradation. Finally, with increased immune cell activity, there is increased IFX proteolysis and phagocytosis. In obesity, particularly with visceral adipose tissue, there is increased TNFα, which also acts as a ‘TNFα sink’. There is also increased systemic proteolytic activity, which increases IFX degradation.

One barrier to achieving therapeutic IFX exposure in ASUC is the multiple mechanisms which increase drug clearance (Figure 1) [24]. Inflamed mucosa express high levels of TNFα which bind the circulating IFX, thereby depleting circulating levels and acting as an “anti-TNFα sink” [25]. Severe epithelial disruption increases faecal IFX loss, and higher early faecal loss correlates with primary non-response [26]. Furthermore, the high numbers of mononuclear cells degrade large amounts of IFX through phagocytosis [27], as well as increasing proteolysis of infliximab [28]. Neonatal Fc receptor-mediated recycling of IFX, a pathway that usually prolongs the drug’s half-life by circumventing usual lysosomal degradation, is also reduced as a result of colonic epithelial damage, as well as increased receptor saturation in high inflammatory states [27]. These reductions in circulating drug can also heighten immunogenicity and predispose to anti-drug antibody formation [29].

The impact of a high inflammatory burden on IFX exposure is exemplified C-reactive protein (CRP) data, where values >50 mg/L are independently associated with lower drug levels [29]. The PREDICT-UC randomised trial evaluated intensified and accelerated induction to overcome these mechanisms of increased IFX drug clearance. Although neither 10 mg/kg nor accelerated dosing achieved statistically significant superiority over standard care, a numerically higher response was seen with 10 mg/kg among patients with CRP ≥50 mg/L [30]. Early PKs were prognostic as day 3 IFX concentrations ≤53.6 μg/mL predicted day 14 treatment failure and ≤57.9 μg/mL predicted 3-month colectomy, and greater faecal IFX loss between days 1 and 7 correlated with better response to 10 mg/kg versus 5 mg/kg [31]. Taken together, faecal IFX clearance appears promising for personalised dosing selection, and current signals support consideration of intensified or accelerated regimens in selected patients. Pharmacokinetic models have attempted to assess personalised dosing strategies of IFX over standard-of-care 5 mg/kg dosing in ASUC. Whilst TITRATE did not meet its primary endpoint, other promising models are being developed and await prospective validation [32,33].

### 3.2. Albumin

Hypoalbuminaemia is distinct from, but often accompanies, severe disease activity. There are several reasons that may explain this. First, albumin is a negative acute-phase reactant with hepatic synthesis suppressed by pro-inflammatory cytokines. In health, approximately 10% of albumin turnover occurs via the gastrointestinal tract, but mucosal inflammation markedly increases this loss [34]. Second, the neonatal Fc receptor normally recycles albumin to prevent degradation, but in highly active disease, this mechanism is impaired, increasing albumin clearance. Finally, patients with active disease often have a worse nutritional status, with reduced intake of amino acids, despite having an increased requirement due to acquired tissue damage, leading to decreased albumin production. These effects are partially counterbalanced by a stimulus for albumin production, often via muscle catabolism, to maintain oncotic pressure [35].

Population PK studies for patients with both CD and UC have demonstrated that higher IFX clearance is associated with low albumin [36]. Trough IFX levels robustly correlate with albumin levels [37]. In moderate–severe UC, albumin <35 g/L associates with lower week 6 IFX levels [29], and in CD, albumin <30 g/L predicts primary non-response (PNR) to anti-TNFα therapy [38]. PREDICT-UC also suggested a numerically higher response to 10 mg/kg (vs 5 mg/kg) among ASUC patients with albumin <25 g/L [30]. The mechanism through which albumin influences infliximab clearance is most likely indirect. Very little infliximab is albumin-bound. Instead, albumin often reflects the degree of mucosal injury, which leads to increased drug clearance, as described in the previous section. Similar effects of albumin levels on ADM PKs have been demonstrated [39].

This is also true for vedolizumab (VDZ), for which population PK studies have shown higher drug clearance with hypoalbuminaemia and states of higher disease activity, such as endoscopic activity or CRP. However, albumin appears to be the strongest covariate affecting PKs [40]. As such, albumin is also included in the clinical decision support tool which can help predict response in CD [41]. This is likely through increased faecal drug loss in active disease [42]. Conversely, whilst population PK studies in ustekinumab, mirikizumab, and risankizumab show a similar direction of effect with albumin and disease activity, the effect size is small to be considered not clinically relevant [43,44,45,46,47]. Janus kinase inhibitors (JAKis), such as upadacitinib, show no effect of albumin [48] or baseline disease severity [49] in PKs. There are no data on the effect of albumin or baseline disease severity on the PK of sphingosine-1-phosphate receptor modulator (S1P) drugs such as ozanimod or etrasimod.

Patients with high inflammatory burden and low albumin may require intensified dosing to achieve therapeutic IFX exposure. Specific criteria for identifying such patients remain poorly defined, and early IFX level or faecal clearance monitoring is not yet practical in routine care. In severe cases, particularly with hypoalbuminaemia, 10 mg/kg dosing and accelerated induction should be considered, especially if early symptom recurrence suggests rapid drug clearance.

### 3.3. Perianal Fistulising Crohn’s Disease

Perianal fistulising CD is challenging to treat and is associated with substantial morbidity and a poor quality of life [50]. Optimising drug exposure is central; anti-TNFα therapy, particularly IFX, remains the standard of care [13]. Cross-sectional data show significantly higher median IFX levels in patients with fistula healing versus active fistulas (15.8 vs. 4.4 µg/mL; *p* < 0.0001), with ≥10.1 µg/mL best associated with healing and a further numerical benefit at ≥20.2 µg/mL [37]. A post hoc analysis of ACCENT-II [51], a multicentre, double-blind, randomised, placebo-control trial of IFX in fistulising CD, demonstrated that higher week 14 IFX levels were independently associated with fistula response. They also defined thresholds with combined maximal sensitivity and specificity for week 14 composite remission of ≥20.2 μg/mL at week 2, ≥15 μg/mL at week 6, and ≥7.2 μg/mL at week 14 [52]. These higher targets may result from the increased local expression of matrix metalloproteinases (MMPs) in perianal fistulae [53], which may cleave and inactivate IFX and ADM [28], resulting in higher serum levels required to overcome this. Accordingly, proactively targeting IFX troughs of at least 7.2 µg/mL is recommended, with higher targets often warranted in complex perianal disease. Similar evidence exists for ADM, where ~12.1 µg/mL optimally associates with complete fistula healing [54]. Whilst for other drugs, such as ustekinumab, drug levels correlating with fistula response have been measured to attempt to define target levels, there is significant heterogeneity between the indicated target levels in different studies in CD, and thus no clear target levels can be set [55,56,57]. There is no data to support different targets or dosing strategies for other drugs.

## 4. Body Composition

### 4.1. Obesity

Bodyweight, body composition, and obesity have long been linked to IBD natural history and treatment effectiveness, including PKs. This is of particular interest as 15–40% of IBD patients are now obese [58]. The widespread use of GLP-1 receptor agonists introduces a modifiable variable: intentional weight loss and shifts in fat/lean mass may alter drug exposure, particularly for weight-dependent dosing and subcutaneous biologics, potentially prompting a re-assessment of TDM targets and strategies [59].

Some evidence suggests that IBD follows a milder natural history in obesity [60], whereas other studies report that obese patients treated with IFX are both more likely to relapse, and do so earlier in their treatment course, than non-obese patients [61]. However, meta-analyses of the pivotal IFX trials (ACCENT-1, SONIC, ACT-1, ACT-2) found no association between obesity and IFX response [62]. Similarly, whilst bodyweight has been associated with higher IFX clearance [36], body mass index (BMI) is not always a reliable predictor of IFX [63].

When considering the mechanism through which obesity might affect IFX drug levels, volume of distribution is one factor to consider. Whilst the weight-based dosing of IFX might be expected to overcome this, there is evidence that the volume of distribution in the peripheral compartment decreases with increasing body mass [64]. This would predict a potential overcompensation of dosing in higher-bodyweight individuals and an undercompensation in lower-bodyweight individuals based on weight-based IFX dosing. However, both weight and BMI are inherently poor ways of defining obesity, as they take no account of the differential effect of body composition, where fat and muscle mass may be more important for the specific effect of obesity.

The effect of BMI and bodyweight is more straightforward in ADM. A prospective cohort analysis nested within the COMBINE trial of anti-TNF monotherapy vs. combination therapy with oral methotrexate in paediatric CD demonstrated patients with BMI > 1 standard deviation above mean had lower drug concentrations compared to patients with normal BMI (median 5.8 vs. 12.8 mg/mL, *p* = 0.02), but this effect was not seen with IFX [65]. In adults, a higher bodyweight was also an independent covariate predicting lower ADM levels in a population PK model developed from data from the SERENE CD and UC trials [66].

One mechanism through which increased biologic drug clearance may occur in obesity is due to increased proteolysis, which is seen in obese individuals [67,68]. Adipose tissue, particularly in obesity, overexpresses TNFα due to macrophage accumulation, creating an “anti-TNFα sink” analogous to ASUC (Figure 1) [69,70]. Site-specific effects are relevant as mesenteric adipocytes produce more TNFα than subcutaneous adipocytes, and patients with CD exhibit a higher intra-abdominal-to-total abdominal fat ratio [71]. “Creeping fat,” a mesenteric fat subtype encasing the bowel, is characteristic of ileal CD, correlates with transmural inflammation, fibrosis, muscular hypertrophy, and stricture formation, and is present in approximately 20% of patients [72,73]. Anti-TNFα outcomes appear worse at both extremes of visceral adipose tissue (VAT) volume (≥3000 cm^3^ and <1500 cm^3^) [74].

Poor response in the patient group with very low visceral adipose tissue volume likely parallels adverse outcomes seen with sarcopenia, where myopenia itself predicts primary non-response to anti-TNFα, malnutrition both worsens outcomes and reflects advanced disease, and low bodyweight leads to increased volume of distribution of IFX in the peripheral compartment [38,64,75]. Whether targeted TDM can offset the adverse impact of high visceral adipose tissue is uncertain. However, non-linear data suggest different trough requirements by very low visceral adipose tissue—steroid-free deep remission and endoscopic remission were associated with 3.9 µg/mL in the lowest two quartiles versus 15.3 µg/mL in the highest two quartiles [76].

Extremes of bodyweight are a significant predictor of PKs in vedolizumab, although the clinical relevance of this is uncertain [40]. Similarly, a post hoc analysis of IM-UNITI investigating ustekinumab in CD demonstrated a BMI ≥ 30 kg/m^2^ significantly predicts lower drug levels but does not predict clinical response [77]. In contrast, thiopurines show no clear relationship between 6-thioguanine (TGN) levels and body composition metrics, including adiposity and total weight [78]. Similarly, although there is a detectable effect of weight on PKs of mirikizumab, risankizumab, JAKis, and S1Ps, the effect size is too small to be deemed clinically relevant [43,45,46,47,48,79].

Integration of tissue volumetrics into treatment target algorithms may be achievable in time. For now, in patients with obesity, targeting higher trough concentrations, or deferring a declaration of treatment failure until higher thresholds are reached, may be guided by clinical assessment of body composition rather than weight or BMI alone.

### 4.2. Pregnancy

**Figure 2:**
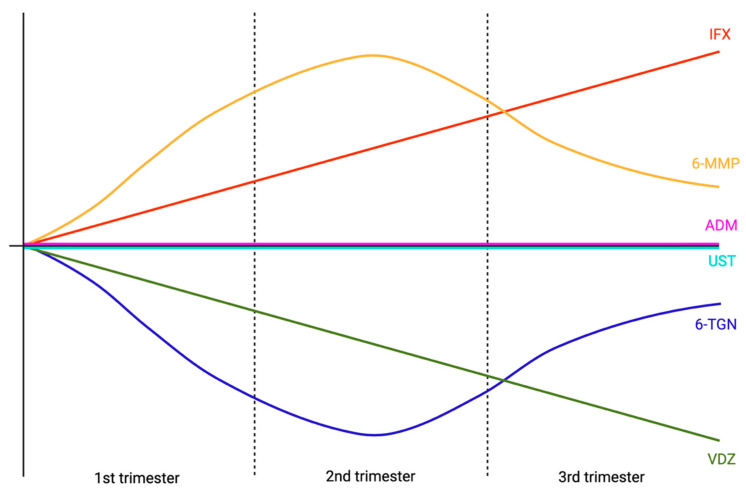
Graphical representation of drug level trends in pregnancy. Throughout pregnancy, infliximab (IFX) levels rise. Adalimumab (ADM) and ustekinumab (UST) levels remain static. Vedolizumab (VDZ) levels reduce throughout pregnancy, correlated with increasing patient weight. 6-thioguanine (6-TGN) levels reduce from baseline to their lowest point in the 2nd trimester before returning towards, but not quite back to, normal. 6-methylmercaptopurine (6-MMP) levels rise during pregnancy, peaking in the 2nd trimester before returning towards, but not quite back to, normal in the 3rd trimester.

Physiological adaptations in pregnancy alter PKs by expanding volume of distribution and modifying metabolic–catabolic pathways [80]. As disease relapse during pregnancy increases the risk of perinatal complications, including pre-term birth and low neonatal birthweight [81,82], appropriate dosing across all trimesters is essential and hinges on how drug levels change over gestation (Figure 2).

IFX levels appear to increase throughout pregnancy trimesters, which remains true even when accounting for the variable changes in BMI, albumin, and CRP, suggesting decreased drug clearance during pregnancy [83,84]. The increase has been calculated as 0.16 µg/mL/week, or 6.4 µg/mL over a 40-week pregnancy [84]. This may be because of increased neonatal Fc receptor expression during pregnancy, which enhances IFX recycling [85]. By contrast, adalimumab shows little change through pregnancy [83,84]. Thiopurines display a different pattern, with 6-TGN levels falling and 6-methylmercaptopurine (MMP) levels increasing in the second trimester before returning back towards pre-pregnancy levels in the third trimester [86]. This has implications for potentially increased hepatotoxicity with reduced drug efficacy during pregnancy. Ustekinumab levels appear stable throughout pregnancy [87,88], whereas vedolizumab levels tend to decline in association with weight gain; the clinical significance is uncertain [84,87]. There is a lack of evidence for the effect of pregnancy on mirikizumab and risankizumab PKs. Whilst the small molecules tofacitinib, filgotinib, upadacitinib, ozanimod, and etrasimod are contraindicated during pregnancy due to teratogenicity, major societies strongly recommend continuing anti-TNFα drugs and thiopurines during pregnancy, as well as other non-anti-TNFα biologics, particularly if active or historically difficult-to-treat disease is present [15,89]. This is because of their well-documented safety in pregnancy, with any detectable risk outweighed by the risk to mother and child of flare after drug discontinuation [90,91,92,93,94].

## 5. Age

### 5.1. Elderly

Ageing IBD cohorts are expanding; in 2010–2014, adults >65 years accounted for 26% of index IBD admissions in the USA [95]. This reflects compounding prevalence—incidence has stabilised, but improved outcomes and survival increase the number of older adults living with IBD [96]. Modelling from Canada, Denmark, and Scotland projects continued growth in the older IBD population through 2023–2043 [97].

Older patients face unique challenges: comorbidities, polypharmacy, frailty, and altered PKs all influence drug disposition and treatment outcomes. Thiopurines and JAKis carry higher risks of adverse events, including infections, cytopenias, and malignancies, particularly in patients over 60 [98,99]. Yet dosing and monitoring data for advanced therapies in this group are sparse because older patients are underrepresented in trials; from 2011 to 2018, those ≥75 years comprised only 1.3% of IBD trial participants [100,101]. Observational studies are therefore the main source of guidance, but heterogeneity in design and reporting limits certainty.

For IFX, available evidence indicates a similar exposure–response relationship in patients ≥65 and younger adults, while serious adverse events and malignancy rise with age independent of IFX exposure [102]. This implies that the same TDM targets can be applied in elderly patients, but interpretation should be set against a higher background risk of adverse events. PK studies of ustekinumab in adult trials suggest no effect of age within the studied range (18–64) [43]. There is no evidence of age affecting PKs of mirikizumab, risankizumab, vedolizumab, JAKis, or S1Ps within studied age ranges of adults. Whilst polypharmacy does not appear to be an issue affecting the PK of biologic drugs, small molecules, which include JAKis and S1Ps, are typically metabolised by Cytochrome P450 (CYP) enzymatic pathways and thus subject to drug–drug interactions. Etrasimod and ozanimod are metabolised by CYP2C8 and thus their levels are increased by CYP2C8 inhibitors, including clopidogrel and gemfibrozil, whilst conversely rifampicin may reduce their levels. Upadacitinib and tofacitinib are metabolised by CYP3A4, and thus their levels are potentiated by inhibitors including ciprofloxacin, clarithromycin, diltiazem, fluconazole, and verapamil. Conversely, carbamazepine, phenytoin, and rifampicin reduce their levels. Filgotinib, however, is an exception that is not metabolised predominantly through CYP enzymatic pathways and thus not subject to drug–drug interactions [103,104].

Future studies could examine whether frailty, sarcopenia, and altered body composition modify PKs in the elderly, and whether higher trough levels carry a different balance of risk and benefit in this population. Defining optimal TDM thresholds in the elderly will likely require pragmatic registry studies that incorporate outcomes such as adverse and serious adverse events, including hospitalisation and functional decline. Until then, application of standard TDM targets in older adults remains reasonable, provided decisions are contextualised within individual comorbidity profiles and vigilant clinical surveillance.

### 5.2. Paediatrics

Paediatric guidelines predate adult guidance and, unlike most adult recommendations, endorse proactive TDM rather than reactive use alone [12,105]. The PAILOT study demonstrated that, when using ADM in paediatric patients with CD, proactive TDM was superior to reactive TDM in achieving steroid free clinical remission [6]. Concentration targets broadly mirror adult targets; PANTS, which included patients ≥6 years, associated remission with 7 μg/mL for IFX and 12 μg/mL for ADM [11].

As in adults, higher IFX targets in perianal fistulising CD have also been suggested, with week 14 trough levels of 12.7 μg/mL seen in responders versus 5.4 μg/mL in the active disease group [106]. Children <10 years appear more prone to subtherapeutic troughs and to require escalation and intensified regimens despite similar baseline characteristics [107]. This may in part be due to increased volume of distribution in the peripheral compartment with low bodyweights [64]. This supports consideration of upfront intensified dosing and/or more frequent proactive TDM in this age group. For ustekinumab, PKs appeared to be related to bodyweight, with patients <40 kg more likely to have lower trough levels despite bespoke weight-based dosing regimens [108]. Other drugs are either not licenced in paediatric populations or have no evidence to suggest altered PKs.

IFX clearance is emerging as an additional TDM metric in paediatrics. In ASUC, higher IFX clearance at baseline and week 26 is predictive of colectomy. Higher IFX induction may overcome this—a median 9.9 mg/kg induction resulted in a 1-year colectomy rate of 2.7% compared to 52% with previous the 5 mg/kg standard-of-care induction [109,110]. Similarly, in CD there is a strong negative association between early IFX clearance and subsequent remission [111]. This is driven in part by ADA formation, which increases IFX clearance even at lower levels. Lower overall (area under the curve) IFX exposure between weeks 0 and 14 predicts ADA development and low-level ADA formation and thus higher IFX clearance may be reversed with dose intensification [112]. Taken together, early assessment of IFX clearance may additionally guide early treatment escalation. Alternatively, more pragmatic upfront intensive dosing strategies may prevent pharmacokinetic and immunogenicity failures with IFX.

Models have also been developed based on population PK studies to inform early IFX dose intensification in both UC and CD, with multi-model approaches appearing to provide incremental benefit over single model approaches in retrospective data sets [113,114]. The upcoming REMODEL-CD clinical trial will prospectively assess standard of-care IFX dosing regimens in paediatric CD versus a novel precision dosing platform which determines the starting dose based on covariates including weight, albumin, and ESR and then sets personalised doses and drug level targets according to the most recent measured drug trough level, CRP, faecal calprotectin (fCal), and disease activity score [115].

## 6. Administration

### 6.1. Thiopurines: Monotherapy or Combination

When used as monotherapy, 6-TGN levels of 235–450 pmol/8 × 10^8^ red blood cells (RBCs) correlate best with response, conferring 3-fold higher remission rates; this range is therefore the standard monotherapy target. Thiopurines are increasingly employed as immunomodulators to curb anti-TNFα immunogenicity, particularly with IFX, a major cause of treatment failure [11]. Observational data suggest that, in combination therapy, lower 6-TGN targets, around 125 pmol/8 × 10^8^ RBCs, adequately predict higher IFX levels and lack of ADA development [116,117]. Targeting a lower level may enable reduced exposure to unnecessary burden of immunosuppression or thiopurine toxicity [118]. Other retrospective data suggest traditional 6-TGN target levels of 235–450 pmol/8×10^8^ RBCs are required to prevent IFX ADA formation [119]. Much larger observational data also suggests higher thiopurine analogue doses (azathioprine ≥2.2 mg/kg, mercaptopurine ≥1.1 mg/kg) may be required to reduce the chance of anti-TNFα loss of response in CD [120]. However, a randomised drug withdrawal trial for IBD patients in remission showed that halving the azathioprine dose had no significant effect on IFX trough levels [121]. Neither of these trials defined 6-TGN targets. In summary, evidence for a specific combination-therapy 6-TGN target is mixed; requirements are likely less stringent than for monotherapy, but no consensus target is established.

### 6.2. Route: Subcutaneous or Intravenous

With intravenous (IV) IFX, serum levels fluctuate widely over the dosing interval (peaks often >100 µg/mL), so true trough sampling is essential for valid TDM [64]. In contrast, subcutaneously (SC) administered ADM displays very minimal drug level variation throughout the treatment cycle such that TDM can be performed at any time [122]. The recent availability of SC IFX provides patients with greater flexibility in administration, reduces the burden on infusion units and associated healthcare costs, and, given its comparable immunogenicity profile to IV IFX in combination with immunomodulators, raises the possibility of monotherapy [123,124,125]. The MINIMISE study is expected to clarify whether SC IFX monotherapy is a viable strategy [126].

Like ADM, SC IFX achieves stable serum concentrations throughout its biweekly dosing interval, meaning TDM can reliably be performed at any time [127]. Serum stability is also seen when switching to biweekly SC VDZ from IV administration [128]. When switching from a median IV IFX dose of 500 mg every 8 weeks to SC IFX 120 mg every 2 weeks, trough levels increase from 8.2 ± 4.5 µg/mL to steady-state levels of 14.5 ± 6.0 µg/mL, without a significant change in steroid-free remission rates [124]. Standard SC IFX dosing has also been shown to maintain remission in patients previously escalated to IV IFX regimens up to 10 mg/kg every 6 weeks [129]. Another study in patients switching to SC who at baseline were receiving a range of IV IFX dosing regimens from 5 mg/kg 8-weekly (52%) to >5 mg/kg 4-weekly (5%) showed a median increase in measured levels from 5.25 µg/mL to between 15.1 and 15.4 µg/mL at week 12–52 post-switch. This study suggested optimal SC IFX targets for clinical and biochemical remission based on receiver operating characteristic analysis of 12.2 μg/mL at week 12 and 13.2 μg/mL week 56 for UC and CD [130]. Different treatment endpoints may require different threshold drug levels in SC IFX, as with IV dosing [131]. A cross-sectional study of UC and CD patients predicted targeting IFX levels ≥12 μg/mL to achieve clinical remission alone, ≥16 μg/mL for combined clinical remission and CRP <5 mg/L, and to achieve deep remission (combined clinical remission, CRP <5 mg/L, and fCal <250 μg/g), targeting ≥20 μg/mL was required. These thresholds are higher than those suggested for IV IFX and should be taken into account when interpreting drug levels following a switch to SC administration.

## 7. Conclusions and Future Directions

TDM is increasingly recognised as a key tool in the precision management of IBD, providing a structured approach to optimise drug exposure, prolong treatment durability, and improve outcomes. Beyond simply guiding dose adjustments, TDM distinguishes PK from mechanistic failure, reduces unnecessary drug cycling, and supports safer, more cost-effective use of anti-TNFα drugs. For thiopurines, metabolite monitoring not only mitigates toxicity but also enhances anti-TNFα persistence by reducing immunogenicity.

Although much of the current evidence remains retrospective or observational, this review highlights patient-, disease-, and treatment-specific factors that shape PKs (Table 1) and target concentrations, with direct implications for clinical practice (Table 2).

States of high inflammatory burden may require higher anti-TNFα doses to counter accelerated clearance [31]. In acute care, TDM utility is limited by long assay turnaround times, particularly outside specialist centres. Hence, upfront accelerated or intensified IFX dosing may be more pragmatic given the favourable safety profile of intensified regimens. Targeting higher trough levels of anti-TNFα may be required in perianal fistulising CD to promote fistula healing [37,54,120]. Similarly, higher trough levels may also be required in patients with higher visceral adipose tissue [76], although the most appropriate metrics to measure and the most practical way to assess this are still to be clearly defined. With increasing obesity in IBD cohorts, prospective trials in this area, particularly for anti-TNFα drugs, would be welcomed. Anti-TNFα levels appear stable during pregnancy; by contrast, thiopurine metabolism shifts in the second trimester (falling 6-TGN and rising 6-MMP) may reduce efficacy and increase toxicity [83,86]. Paediatric patients are less likely to achieve target anti-TNFα drug levels [107], and proactive TDM is well supported [6], warranting vigilant monitoring and consideration of early intensive dosing within the paediatric population.

Further progress in TDM for special situations should be derived from studies that explore context-specific exposure targets. ASUC, perianal fistulising CD, pregnancy, paediatrics, and obesity or body composition are the settings where standard targets most often fall short. Here, prospective work should relate early PKs, including faecal infliximab in ASUC, to hard outcomes such as colectomy-free survival, fistula healing on MRI, and steroid-free remission. Subcutaneous infliximab also needs clear induction and maintenance targets, with practical rules for adjustment after switching from IV, so that any time sampling can translate into confident dose changes.

Evidence for non-anti-TNFα therapies remains uneven, and concentration response relationships are weaker. Vedolizumab saturates α4β7 receptors at very low drug levels [132], and different studies suggest very different thresholds, limiting their applicability [133,134,135]. Whilst an exposure–response relationship is well documented for ustekinumab [136], the inability to recapture response after dose optimisation in CD questions the utility of TDM here [137]. There is limited data regarding TDM in mirikizumab and risankizumab to date. Whilst there is a clear dose–response relationship for JAKis, plasma concentrations do not appear to be related to response [138,139]. Ozanimod and etrasimod also do not have a clearly defined exposure–response relationship.

Assay harmonisation is also important, with drug-tolerant ADA methods, cross-platform calibration, and clear reporting of sampling timing all needed to allow results to be compared reliably across centres. Turnaround times must be shortened through point-of-care testing, home sampling, and integration with electronic records, so that results can inform same visit decisions in both specialist and non-specialist settings. Clinical utility will also depend on the development of dosing tools that combine routine clinical variables such as albumin, CRP, phenotype, route, body composition, co-therapy, and prior levels to suggest adjustments and highlight risk of immunogenicity or loss of response. Pragmatic trials with cost–benefit analyses are also needed to establish which proactive, reactive, or hybrid approaches to TDM deliver the greatest benefit and value across different healthcare systems. Ultimately, the goal is a comprehensive PK dashboard or model-informed precision dosing tool that integrates patient characteristics, disease features, route and co-therapy, and treatment intent to define both starting doses and subsequent TDM targets. Although recent prospective trials have not achieved their primary endpoints [32], whilst further trials are awaited, recognition of the individual factors influencing PKs can already support more personalised, case-by-case decision making in UC and CD.

## Figures and Tables

**Table 1:** Summary of factors affecting pharmacokinetics.

Scenario	Drug	Factors Affecting Pharmacokinetics	
**ASUC**		**Negative factors**	
	IFX	Increased faecal drug loss ^†^ TNFα sink ^a,†^ Increased proteolysis ^†^ Decreased neonatal Fc receptor-mediated drug recycling ^†^ ADA formation with low drug levels ^†^	
**Hypoalbuminaemia**		**Negative factors**	
	IFX and ADM	Indirect marker of high inflammatory burden Increased faecal drug loss ^†^ TNFα sink ^a,†^ Increased proteolysis ^†^ Decreased neonatal Fc receptor-mediated drug recycling ^†^ ADA formation with low drug levels ^†^	
	VDZ	Indirect marker of high inflammatory burden Increased faecal drug loss ^†^	
**Perianal fistulising CD**		**Negative factors**	
	IFX and ADM	Increased local fistula expression of MMPs, which degrade mAbs	
**Obesity**		**Negative factors**	**Positive factors**
	IFX and ADM	TNFα sink ^a,†^ Increased proteolysis	Decreased peripheral volume of distribution at higher bodyweights ^‡^
	VDZ	Volume of distribution at extremes of bodyweight ^b,†^	
**Pregnancy**		**Negative factors**	**Positive Factors**
	IFX		Increased neonatal Fc receptor recycling ^‡^
	Thiopurine	Increased 6-MMP, decreased 6-TGN	
	VDZ	Increased volume of distribution with increasing bodyweight ^†^	
**Elderly**	No specific differences		
**Paediatrics**		**Negative factors**	
	IFX and ADM	Children <10 more prone to subtherapeutic dosing Increased peripheral volume of distribution in lower bodyweights ^†^	
**Route: SC or IV**		**Positive factors**	
	IFX	Slower absorption, more stable exposure profile Potentially reduced immunogenicity ^‡^	
	VDZ	Slower absorption, more stable exposure profile	
**Co-administration with anti-TNFα**		**Positive factors**	
	Thiopurine	Reduced anti-TNFα immunogenicity ^‡^ Increased anti-TNFα drug levels	

^†^ Factor that decreases drug exposure. ^‡^ Factor that increases drug exposure. ^a^ TNFα refers to the effect of excess TNFα binding to and thereby neutralising the anti-TNFα drug. ^b^ There is no clear evidence that increased bodyweight reduces clinical response as a result.

**Table 2:** Summary of therapeutic drug monitoring and drug dosing recommendations.

Scenario	Infliximab	Adalimumab	Thiopurine
**General:** **post-induction**	Week 14: 7–10 μg/mL	Week 16: 8–12 μg/mL CD ≥ 12 μg/mL	6-TGN 235–450 pmol/8 × 10^8^ RBCs
**General:** **maintenance**	5–10 μg/mL	8–12 μg/mL CD ≥ 12 μg/mL	6-TGN 235–450 pmol/8 × 10^8^ RBCs
**ASUC**	Consider 10 mg/kg dosing +/− accelerating induction in select patients, particularly in hypoalbuminaemia	-	-
**Hypoalbuminaemia**	Consider 10 mg/kg dosing or early TDM to direct dose escalation	Consider 40 mg weekly dosing or early TDM to direct dose escalation	No evidence for different targets
**Perianal fistulising CD**	Consider ≥10 μg/mL	≥12 μg/mL	No evidence for different targets
**Obesity**	High visceral adipose tissue: consider higher target	High visceral adipose tissue: consider higher target	No evidence for different targets
**Pregnancy**	Levels rise throughout pregnancy No evidence for different targets	Levels stable throughout pregnancy No evidence for different targets	2nd trimester: 6-TGNs drop, 6-MMPs rise 3rd trimester: return towards baseline
**Elderly**	No evidence for different targets	No evidence for different targets	No evidence for different targets
**Paediatrics**	Proactive TDM—no evidence for different targets Age <10 more likely to have sub-optimal levels, consider intensified dosing and early proactive TDM	Proactive TDM—no evidence for different targets Age <10 more likely to have sub-optimal levels, consider intensified dosing and early proactive TDM	No evidence for different targets Adherence is main factor affecting levels
**Route: SC or IV**	SC switch from typical IV regimens may raise drug levels by 6–10 μg/mL Consider ≥12–20 μg/mL	-	-
**Co-administration with anti-TNFα**	-	-	6-TGN >125 pmol/8 × 10^8^ RBCs may be sufficient

## Data Availability

No new data were created or analysed in this study. Data sharing is not applicable to this article.

## Abbreviations

The following abbreviations are used in this manuscript:

ADM	Adalimumab
ASUC	Acute severe ulcerative colitis
CRP	C-reactive protein
CYP	Cytochrome P450
fCal	Faecal calprotectin
GLP-1	Glucagon-like peptide-1
IV	Intravenous
mAb	Monoclonal antibody
PK	Pharmacokinetic
RBC	Red blood cell
SC	Subcutaneous
TDM	Therapeutic drug monitoring
TNFα	Tumour necrosis factor alpha
UST	Ustekinumab
VDZ	Vedolizumab
6-MMP	6-Methylmercaptopurine
6-TGN	6-Thioguanine
